# Semantic impairment disrupts perception, memory, and naming of secondary but not primary colours.

**DOI:** 10.1016/j.neuropsychologia.2015.01.010

**Published:** 2015-04

**Authors:** Timothy T. Rogers, Kim S. Graham, Karalyn Patterson

**Affiliations:** a Department of Psychology, University of Wisconsin-Madison, 524 WJ Brogden Hall, 1202W Johnson Street, Madison, WI 53706, USA; b Wales Institute of Cognitive Neuroscience, School of Psychology, Cardiff University, Tower Building, 70 Park Place, Cardiff CF10 3AT, UK; c University of Cambridge, Department of Clinical Neuroscience, Neurology Unit, Addenbrooke’s Hospital, Cambridge CB2 0SZ, UK; d MRC Cognition and Brain Science Unit, 15 Chaucer Road, Cambridge CB2 7EF, UK

**Keywords:** Semantics, Colour perception, Dementia

## Abstract

To investigate how basic aspects of perception are shaped by acquired knowledge about the world, we assessed colour perception and cognition in patients with semantic dementia (SD), a disorder that progressively erodes conceptual knowledge. We observed a previously undocumented pattern of impairment to colour perception and cognition characterized by: (i) a normal ability to discriminate between only subtly different colours but an impaired ability to group different colours into categories, (ii) normal perception and memory for the colours red, green, and blue but impaired perception and memory for colours lying between these regions of a fully-saturated and luminant spectrum, and (iii) normal naming of polar colours in the opponent-process colour system (red, green, blue, yellow, white, and black) but impaired naming of other basic colours (brown, gray, pink, and orange). The results suggest that fundamental aspects of perception can be shaped by acquired knowledge about the world, but only within limits.

## Highlights

• Study of colour perception and cognition in patients with semantic dementia.
• Colour discrimination is normal but categorization is impaired.
• Patients and controls show opposing accuracy patterns in memory and perception.
• Naming is more impaired for secondary than primary colours.

## 1 Introduction

To what extent does our acquired knowledge about the world influence how we perceive it?

The study of colour cognition—our ability to perceive, categorize, remember, and name colours—has long provided a test-bed for this central and controversial question. Subjectively, colours feel as though they are of the world—attributes of surfaces that are sensed directly and automatically from the environment. We do not feel as though we have learned how to perceive “green”, nor does it seem that the way “green” looks should depend upon the language we speak or the knowledge we have acquired about the world. Foundational research in human colour cognition has tended to bear out such intuitions (Berlin and Kay, 1969): colour information is thought to be carried along three neurophysiological channels, each corresponding roughly to an independent psychophysical dimension—a red/green channel, a blue/yellow channel, and a black/white (ie light/dark) channel—and perceived colours are thought to arise from patterns of stimulation across the three channels (Hurvich and Jameson, 1957; Solomon and Lennie, 2007), subject to corrective mechanisms that preserve colour constancy under different conditions of illumination. It is commonly thought that the degree to which different colours appear similar is determined predominantly by such “bottom-up” stimulation (Bornstein, 1973), that the words we use to refer to colours are strongly constrained by such perceptual structure (Berlin and Kay, 1969; Heider, 1972; Kay, 2005), and that the perceived similarities amongst various colours are not amenable to change through learning, experience, or language (Berlin and Kay, 1969).

Over the past two decades, however, this standard view has been challenged on several fronts, including a sustained critique of the methodology and the interpretation of results from seminal studies (Saunders and van Brakel, 1997), failure to replicate some of these early results in new studies employing adapted methods (Roberson et al., 2000), and data from new cross-cultural (Davidoff et al., 1999; Roberson, Davidoff et al., 2004), psychophysical (Goldstone, 2006; Ozgen and Davies, 2002) and developmental studies (Roberson et al., 2004). Much of this new research suggests that the ability to discriminate and to discern similarities amongst colours varies considerably depending upon the language one speaks or the way one has learned to categorize colours (Goldstone, 2006; Ozgen and Davies, 2002; Roberson et al., 2000). This research has itself been subject to intense critical scrutiny (Kay, 2005; Kay and Regier, 2003, 2006), leading to heated debate regarding the validity of the standard theory. Saunders and van Brakel (1997), for instance, write “In each subfield… there are respected local experts who have already made devastating criticisms. What we have here is an apparently coherent study of the type that most scientists would like to believe in, but is seriously entertained only because people over-charitably assume that the parts in which they are *not* experts are sound” (Saunders and van Brakel, 1997, p. 169). The controversy has re-opened a very basic question for cognitive science: To what extent does the structure of the world shape our minds, and vice versa?

We report a new source of evidence addressing this question through the study of colour cognition in a group of neurological patients who are progressively losing their knowledge about the world. *Semantic dementia* (SD) is a degenerative disease that destroys anterior regions of temporal cortex bilaterally, producing significant impairments to language production and comprehension, recognition, and the ability to infer properties of objects and events on the basis of their meaning (Warrington, 1975; Saffran and Schwartz, 1994; Lambon Ralph et al., 1999; Patterson and Hodges, 2000; Snowden, Thompson and Neary, 2004; Patterson, Nestor, and Rogers, 2007; Guo et al. 2013). Despite these disabilities, patients with SD perform well on tests of visual perception including colour discrimination (Barense et al., 2010; Lee et al., 2007) and on most other tests of non-semantic cognitive functioning (Barense et al., 2010; Hodges et al., 1998; Hodges et al., 1999; Rogers et al., 2006). Moreover, until late in the disease process, the selective brain atrophy that produces the disorder is largely circumscribed within anterior parts of the temporal lobes, typically sparing the posterior–inferior cortical regions such as V4 thought to support colour perception (Acosta-Cabronero et al., 2011; Guo et al., 2013; Mion et al., 2010; Mummery et al., 2000; Nestor et al., 2006). SD thus provides a unique opportunity to investigate whether and how colour perception and cognition are affected when general knowledge about the world degrades.

The few previous studies of colour cognition in SD suggest that, though knowledge of colour names deteriorates, other aspects of colour cognition can be spared. Two papers have reported progressive colour anomia proportionate to the general degree of semantic impairment (Haslam et al., 2007; Rogers, Patterson and Graham, 2007), though a third reports that colour naming can be normal in SD (Robinson and Cipolotti, 2001). Discrimination between even very similar colours appears unaffected in SD (Barense et al., 2010; Lee et al., 2007), and one study has found preserved ability to sort colour chips into categories, coincident with progressive colour anomia, in a single case of SD (Haslam et al., 2007). To our knowledge, however, no prior work has systematically evaluated multiple aspects of colour cognition in a case series of SD patients.

In the current work, we assessed colour discrimination, categorization, perception, memory, and naming in case series of patients with SD. The results establish a previously undocumented disorder of colour cognition in this group with strong implications for theories about the interaction between knowledge and perception.

## 2 Experiment 1: Categorization and discrimination in an oddball task

Previous research has shown that even patients with advanced SD can successfully discriminate subtly different colours (Barense et al., 2010; Lee et al., 2007). Colour perception, however, also entails the ability to discern the similarities among discriminably different shades of the same colour category. Experiment 1 assessed both colour discrimination and categorization in SD using an “oddball detection” procedure adapted from that employed in the earlier work with SD.

### 2.1 Participants

Ten patients and 20 healthy age-matched controls participated in the study. One patient was unable to complete the task due to fatigue. All patient participants were diagnosed according to consensus and local criteria for SD; all had MRI-confirmed cortical atrophy concentrated in the anterior temporal lobes (as assessed by a senior neurologist); and all showed the characteristic pattern of impaired performance on verbal and non-verbal tests of semantic memory, together with generally good performance on other aspects of cognition including visuo-spatial perception and colour discrimination. The results of standard neuropsychological assessments for the patients are shown in Table 1. All patients reported having normal colour vision prior to the onset of their disease. Control participants were recruited from the volunteer subject panel at the MRC Cognition and Brain Sciences Unit in Cambridge and were matched to the patient participants for age.

### 2.2 Hardware and stimuli

The oddball-detection task was programmed on a computer using Visual Basic. Patients were tested in their own homes using a Dell Latitude 110L laptop PC with an XGA liquid-crystal display (LCD) set up in a well-lighted room. Control participants were tested either on a desktop computer with a similar LCD display set up in a well-lighted room at the MRC-CBU, or on the same laptop as the patients. The performance of controls tested with the laptop versus the desktop computer did not differ significantly in any task so controls were treated as a single group. Details regarding the nature of the liquid-crystal display and control of colours with this technology, including parameters used to generate stimuli in this and the next experiment, are provided in Appendix I. Briefly, the display controls three “liquid crystal” filters at every pixel, each controlling the intensity of light at a given peak wavelength, with peaks around 450 nm (blue), 550 nm (green), and 650 nm (red). Colours are specified by setting the states of these filters to a value between 0 (filter completely blocks light at target frequencies) to 255 (filter allows complete passage of light at target frequencies). Colour coordinates are specified in this parameter space.

### 2.3 Task

The task was modeled on prior work by other researchers where the goal was to measure discrimination and categorization (e.g. Petzold and Sharpe, 1998; Roberson et al. 1999). On each trial, participants viewed four coloured squares on a computer display and were instructed to click on the square that was a different colour from the rest.

### 2.4 Design

The test included 18 Discrimination and 18 Categorization trials, presented on a computer screen in mixed random order. In Discrimination trials, 3 of the 4 squares were identical in colour whereas the “oddball” differed subtly. In Categorization trials, our goal was to create arrays of discriminable colours with one item reliably judged by healthy controls to belong to a different category from the other three, and with the task equal in difficulty to the Discrimination trials. Thus we aimed to create arrays in which the different options were perceptually variable and discriminable from one another, with an out-of-category oddball that was not too perceptually distant from distractor items. To this end we paired each Categorization array with one Discrimination array and ensured that all items in the Categorization array were more distant from one another in the colour parameter space described earlier than were the discrepant items in the paired Discrimination display. We further ensured that the oddball colour was equally distant from the distractor colours in this parameter space. In a pilot study with healthy age-matched controls using 20 arrays of each type, we selected a subset of 18 pairs of Discrimination/Categorization arrays closely matched for accuracy. Though the colour space used to construct arrays is not psychophysically controlled for perceptual distance, this approach ensured that the Discrimination and Categorization tasks were equally difficult for healthy controls. It also ensured that, among participants who perform well on Discrimination trials, good performance on the Categorization trials entails grouping discriminably different colours together into categories.

### 2.5 Results and discussion

**Fig. 1:**
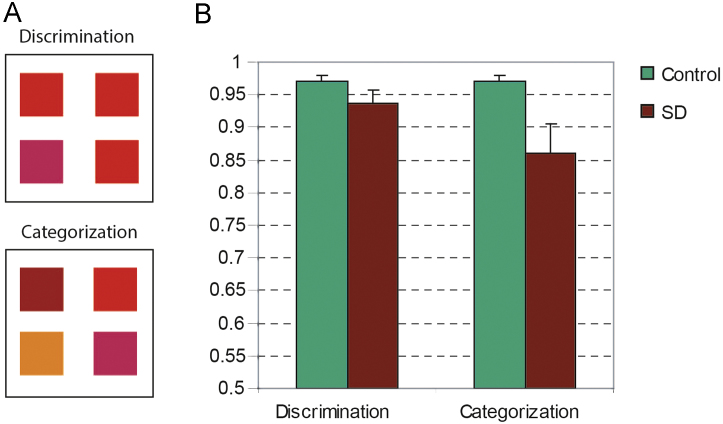
Stimuli and performance in the oddball task. (A) Examples of stimuli used in the Discrimination (top) and Categorization (bottom) trials of the oddball-detection task. In both cases the oddball is in the bottom left of the display, though in the experiment the location of the oddball varied at random. (B) Mean and standard error of the proportion correct across controls and patients for the two trial types. Figures appear in colour in the online version of this article.

The most severely impaired patient (ATh) was unable to complete the task. Of the remaining nine patients, all but one (ATe) performed within the control range for the Discrimination trials. As a group, patient accuracy did not differ reliably from controls (mean for controls=0.97; mean for patients=0.92; *p*=n.s. for 1-tailed between-subjects *t*-test; Fig. 1b). Of the 8 patients who performed normally on Discrimination trials, half performed more than 2 standard deviations below the control mean on Categorization trials, and accuracy was reliably poorer for the whole patient group in this condition (control mean=0.97; patient mean=0.86; *p*<0.001 one-tailed *t*-test). Whereas controls were equally accurate for Discrimination and Categorization trials, patients were reliably worse on Categorization trials (*p*<0.03 two-tailed paired-samples *t*-test). The results thus suggest that the ability to discern differences in colour is largely intact in SD, but the ability to group different colours together into categories is impaired.

## 3 Experiment 2: Perception and memory in a match-to-sample task

It is possible that the categorization impairment illustrated in Experiment 1 arises from the anomia that is a presenting feature of SD. For instance, controls may perform well by naming all colours in the display and choosing the item with a different name—a strategy unavailable to participants who cannot name all the colours. Experiment 2, therefore, assessed perception and short-term memory for colours using a match-to-sample paradigm that does not require covert naming for good performance.

The motivation for this study stems from prior work showing that, despite good performance on tests of visual perception and of short-term memory for arbitrary materials, the ability to retain visual representations of everyday objects over a short interval is degraded in SD (Bozeat et al., 2003; Lambon Ralph & Howard, 2000; Patterson and Erzinclioglu, 2008; Rogers, Lambon Ralph, Garrard, et al., 2004). For instance, such patients can copy line-drawings of familiar objects adequately when directly observing them, but are impaired when asked to reproduce the drawing from memory after a ten‐second span. Moreover, the degraded images produced by patients after a delay are not arbitrary but highly systematic: visual properties that characterize the item’s broad conceptual domain—such as the legs, eyes, and mouths of animals, the wheels on vehicles, or the handles of tools—are largely preserved, or even incorrectly added in, as when the patient depicts both forelegs and hindlegs in a drawing of a duck. Properties that individuate semantic neighbours—such as the humps on camels, the udders of cows, or the slots of a toaster—are very frequently omitted. Thus the patients appear able to retain only the most prototypical attributes of familiar visually-presented items over a short delay. In the current task, we assessed whether the same is true of short-term memory for colours.

### 3.1 Participants

The same 10 patients and 20 healthy age-matched controls from Experiment 1 completed the experiment.

### 3.2 Materials and procedure

The match-to-sample task was programmed on a computer using Visual Basic. Patients were tested in their own homes using the same laptop PC set up in a well-lighted room. Control participants were tested either on a desktop computer with a standard LCD display set up in a well-lighted room at the MRC-CBU, or on the same laptop as the patients. The performance of controls tested with the laptop versus the desktop computers did not differ significantly in any task so controls were treated as a single group. Colour parameters were specified as noted in Experiment 1 and further explained in the Appendix.

### 3.3 Task

**Fig. 2:**
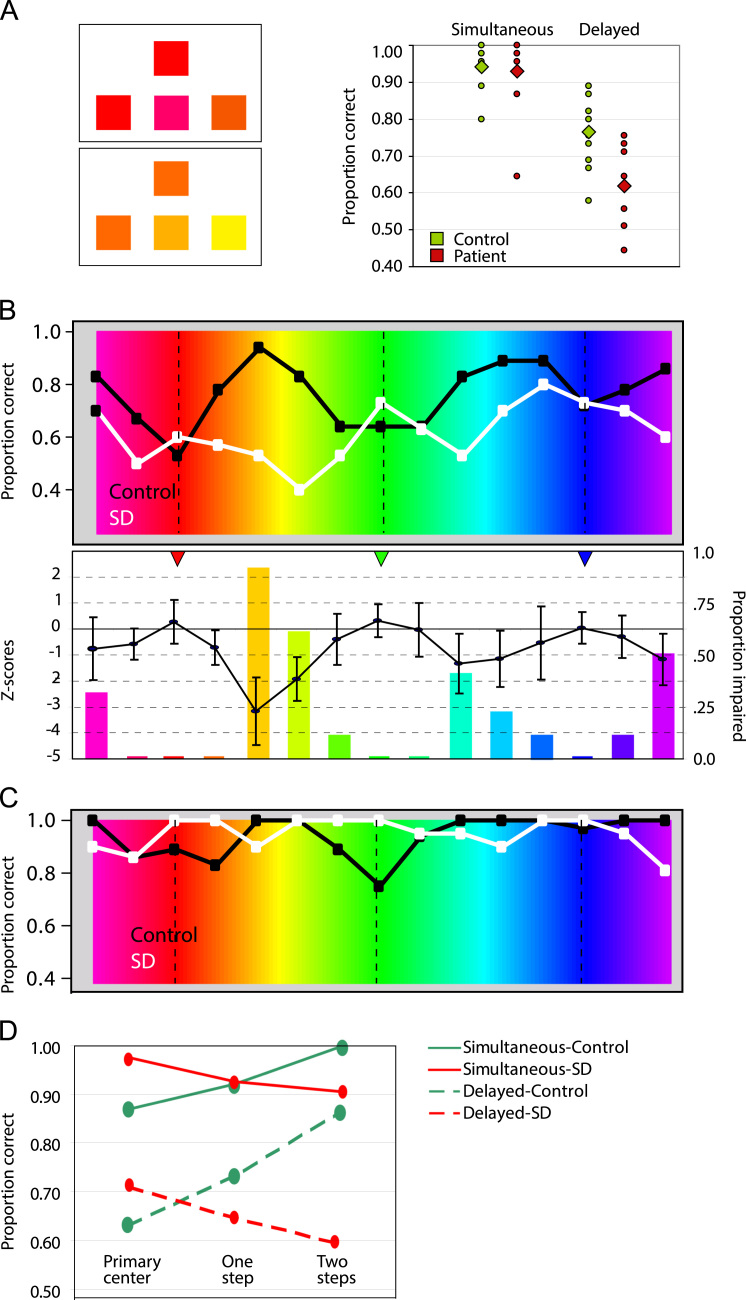
Stimuli and performance in the match-to-sample task. (A) Examples of stimuli (left) and overall performance (right) in the match-to-sample task. The top example has a target at pure red (leftmost choice) and distractors 12 degrees to the left and right of the target. The bottom example has a target at 24° with distractors at 36 and 48°. Dots on the plot indicate the accuracy of individual controls (green) and patients (red) in simultaneous and delayed conditions; diamonds indicate the respective means. Because some patients had identical scores there are fewer points shown than patients. (B) Top: Mean proportion correct in the delayed condition at each test point across the spectrum for controls (black) and patients (white). Bottom: The line shows means and 95% confidence intervals of the Z-scores for patient accuracy at each test point plotted against the left scale. The bars show the proportion of patients with significantly impaired performance at each test point plotted against the right scale. Triangles at the top indicate the location of the red, green, and blue colour centers. (C) Mean proportion correct in the Simultaneous condition at each test point for controls (black) and patients (white). D. Mean performance at the primary (red, green, blue) colour centers, and at one step (24°) and two steps (48°) from these, for patients and controls in simultaneous and delayed conditions. (For interpretation of the references to color in this figure legend, the reader is referred to the web version of this article.)

On each trial participants viewed a coloured square (the sample) at the top of a computer screen, with a *target* square (identical in colour to the sample) and two *distractor* squares (neighboring colours on the spectrum) appearing in random order below (Fig. 2a; left-hand items along the bottom are identical matches in this example while the other two options are distinct neighbors on the spectrum). From the 3 lower squares, participants were instructed to select the one that exactly matched the sample. The experiment began with three very easy practice trials to ensure participants understood the task.

### 3.4 Design

In the Simultaneous condition, all 4 squares appeared together on the screen against a white background, so performance measured the ability to detect that target and sample are identical and that target and distractors are different. In the Delayed condition, the sample appeared for 1 s against a white background, then was replaced by a blank white screen for 3 s after which the 3 options appeared, so performance here also assessed short-term memory for the sample colour.

A fully saturated and luminant spectrum was generated using the procedure described in Appendix 1. From this spectrum we selected 15 points to be sample colours, beginning with the red center (filter at 650 nm fully open and other filters fully closed) and spaced at regular intervals in the filter parameter space indicated by the points on Fig. 2b. Distractor colours included these 15 points along the spectrum plus an additional 15 selected from the midpoints between the samples in the filter parameter space. The samples include instances where each filter is fully open and the other two are fully closed; we will refer to these as the red (650 nm filter open), green (550 nm filter fully open) and blue (450 nm filter fully open) colour centers respectively. Each sample appeared in 3 separate trials—once with two distractors that were immediate neighbours toward the red end of the spectrum, once between the two adjacent distractors, and once with two distractors that were immediate neighbours toward the blue end. Thus the full test included 45 trials in each of the Simultaneous and Delayed conditions.

We note that distances in the filter parameter space are not psychophysically normalized, so that a fixed distance in this space may be easier to perceive or remember in some parts of the spectrum than others. For this reason, we take the behavior of the healthy controls at each sample point as the normative behavior against which to compare the patient data. The central question is whether the controls and patients behave similarly or differently in the Immediate and Delayed conditions, both in their overall performance across colour samples and at each sample point across the spectrum.

### 3.5 Results and discussion

In the Simultaneous condition, all patients except the most severely impaired case (ATh) showed performance within the normal range (right side of Fig. 2a). As a group the patients did not differ significantly from controls on this condition (control mean=0.94, patient mean=0.93, *p*=n.s. for one-tailed *t*-test). In the delayed condition, 4 of 10 patients performed below the control range, and as a group the patients were significantly impaired in this condition (control mean=0.76, patient mean=0.62, *p*<0.001 one-tailed *t*-test).

This impairment did not, however, affect all colours equally. Fig. 2b shows, for the Delayed condition, the average proportion correct for patients and controls at each test point. Controls performed least well at the red, green and blue centers, and best for colours falling near the midpoints defined above. (Note that the exact midpoints between colour centers did not appear as samples; instead samples appeared bracketing either side of each midpoint). Patients with SD showed the reverse saw-tooth pattern, performing *best* in the red, green, and blue centers, and *worst* in the intermediate regions.

For a quantitative assessment of these data, we computed the control mean accuracy and variance at each target point. From these we calculated Z-scores for each patient’s accuracy at each target point, then plotted confidence intervals for the mean Z-scores across patients. The results are shown in the bottom panel of Fig. 2b (left-hand axis): Patient performance was normal at the red, green and blue centers (mean Z-scores of 0.27, 0.31 and 0.03, respectively), but fell significantly below normal in the samples adjacent to the midpoints. Fig. 2b also shows a histogram indicating what proportion of the patient group was significantly impaired at each test point (right-hand axis). At the red-green midpoint, 90% of patients were impaired; at the green–blue midpoint, 40% were impaired, and at the blue-red midpoint, 50% were impaired. No patient showed significant impairment at the red, green, or blue centers themselves.

Following these observations, we re-analyzed data from the Simultaneous condition. Our original analysis showed normal accuracy in the patient group collapsed across all colours. In the re-analysis, we computed the average proportion correct at each sample point along the spectrum for patients and controls (Fig. 2c). This analysis revealed a similar pattern to the Delayed condition: control performance showed small troughs in the red, green, and (less dramatically) the blue centers, with ceiling performance in between these regions, whereas patients showed ceiling performance in red, green, and blue centers, with small troughs between these. We could not compute Z-scores for the patients, as all controls performed at ceiling at many test points (which therefore had no variance). Instead we collapsed the data by computing average performance, for controls and patients, (i) in each of the Simultaneous and Delayed conditions, and (ii) at each of three sample steps from the red, green, and blue centers, that is, at the colour centers themselves, and at one and two sample steps away from these. These data are shown in Fig. 2d for both Simultaneous and Delayed conditions. Analysis of variance for the control data so tabulated revealed i) a main effect of task, with performance much better overall in Simultaneous relative to Delayed conditions (*F*(1,22)=45.2, *p*<0.001), (ii) a main effect of the sample steps from the colour center (*F*(2,22)=23.7, *p*<0.001), with worst performance at the colour center and best at the furthest step, and (iii) no interaction between these (*F*(2,22)=1.1, *p*=n.s.). The same analysis of patient performance showed (i) a main effect of task with performance much better in Simultaneous than Delayed conditions (*F*(1,18)=70.0, *p*<0.001), and (ii) a main effect of distance (*F*(2,22)=3.7, *p*<0.05), with performance at the red, green and blue colour centers significantly *better* than performance at colour samples one and two steps away (*p*<0.04 and *p*<0.03 for contrast to 1-step and 2-step distances, respectively). As with the control data, the interaction between these factors was not significant (*F*(2,18)=0.3, *p*=n.s.). In other words, where controls were better at perceiving and remembering colours in regions between the colour centers, patients showed the reverse pattern.

## 4 Experiment 3: Naming polar and non-polar colours.

Experiment 2 revealed preserved perception and short-term memory in SD for colours near red, green, and blue centers (where controls were weakest), but impaired perception and memory for regions between these areas (where controls were strongest). What accounts for this pattern? One possibility is that the knowledge impairm 4 ents in SD do not affect perception or memory for the “poles” of the opponent-process colour space, but do degrade perception and memory for colours in between these poles. Unfortunately Experiment 2 does not fully address this possibility, for two reasons. First, the stimuli used in this study did not sample sufficiently finely to discern how perception and memory fare in the transition from red to green, which includes the primary colour yellow. Second, the sampling strategy employed only assessed chromatic hues; it was not possible to assess perception and memory for the achromatic polar colours black and white.

Experiment 3 thus sought to test the hypothesis―that polar colours are preserved in SD―in a different way, by assessing naming accuracy for polar (primary) and non-polar (secondary) colours. In prior work, we have shown that colour naming degrades in parallel with object naming in this patient population (Rogers et al., 2007). In this study, we reanalyzed those naming data by considering basic colour naming performance separately for primary versus secondary colours.

### 4.1 Participants

Twelve patients with SD participated in the experiment. All were diagnosed according to consensus and local criteria for SD; all had MRI-confirmed cortical atrophy concentrated in the anterior temporal lobes of the brain (as assessed by a senior consultant neurologist); and all showed the characteristic pattern of impaired performance on verbal and non-verbal tests of semantic memory, together with generally good performance on tests of other aspects of cognition including tests of visuo-spatial perception and colour discrimination. Performance on standard neuropsychological assessments is shown in Table 2. Control data were not collected as controls routinely perform at ceiling with these ten basic colour words.

### 5.2 Materials and procedure

Participants were handed 10 crayons in random order, and were asked to name the colour of each. Responses were recorded on a scoresheet by the experimenter. The crayons included the 6 primary or “polar” colours (white, black, red, green, blue and yellow) and four secondary or “non-polar” colours (brown, gray, pink and orange). We first tabulated, for each of the 10 colours, the total proportion of patients who produced the correct name. We then contrasted mean performance for the 6 polar colours with the 4 non-polar colours in the set.

### 5.3 Results and discussion

In any naming task, the frequency of the target name is a strong predictor of accuracy in SD. It is therefore important to consider the relative frequencies of the 10 basic colour terms assessed. We first considered word frequencies from the classic corpus of Kucera and Francis (1967) which, though small by today’s standards, we take to represent word frequencies at a point in time when our participants were young adults. We also considered word frequency counts from the British National Corpus (BNC), a much larger and more recent corpus specific to the UK where this work was conducted, and drawn from samples of both written and spoken text (Leech et al., 2001). The log frequencies for the 10 basic colour terms assessed are highly correlated in these two corpora (*r*^2^=0.81), but have a somewhat different rank ordering. We thus considered each corpus separately.

**Fig. 3:**
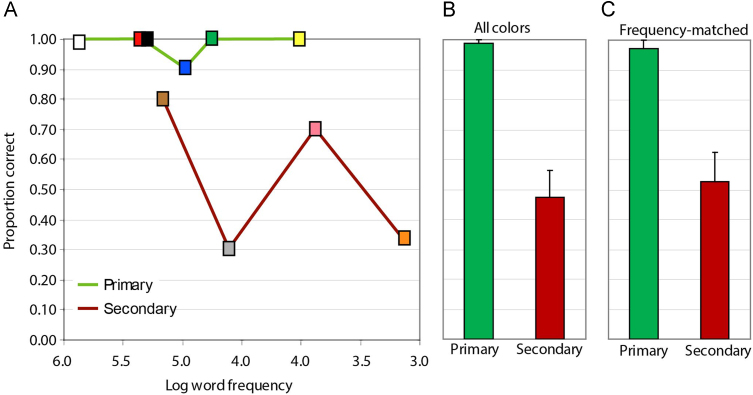
Performance in the naming task. Top left: Proportion of patients who named each colour correctly plotted against log word-frequency from the Kucera and Franics norms, for primary colours (green line) and secondary colours (red line). The colour of each square indicates which colour-name is plotted. Top right: Mean and standard errors across patients for naming of primary and secondary colours considering all 10 colours or just 3 frequency-matched items (from the Kucera and Francis norms) in each condition. Bottom left: Proportion of patients who named each colour correctly plotted against log word-frequency from the British National Corpus (BNC) norms, for primary colours (green line) and secondary colours (red line). Bottom right: Mixed-effects model predicted accuracy for naming primary (green) and secondary (red) colours, as a function of degree of anomia (as assessed by object naming). Lines indicate expected performance at the mean log word frequency across colour names from the BNC norms. Shading indicates the range of expected performance from the lowest to the highest colour word frequency. (For interpretation of the references to color in this figure legend, the reader is referred to the web version of this article.)

The top row of Fig. 3 shows the mean proportion of patients who produced the correct name for each colour, plotted against the colour word frequency according to the corpus of Kucera and Francis (1967). Regardless of word frequency, patients performed at or near ceiling for all 6 primary (i.e. polar) colours. In contrast, performance for secondary (non-polar) colours was impaired, with the most frequent secondary colours (gray and brown) still substantially worse than even the lowest-frequency primary colour (yellow). Thus mean performance across patients was reliably worse for secondary than for primary colours, whether tabulated across all tested colours or subsets matched for word frequency in this corpus (*p*<0.001, 2-tailed paired-samples *t*-test, for both comparisons).

The bottom row shows the same data plotted against the log frequencies derived from the BNC. The results are qualitatively similar, but the two most frequent secondary colour terms according to Kucera and Francis, brown and gray, are substantially less frequent in the BNC corpus. Thus only one primary colour word, yellow, can be matched in frequency to secondary colour words in this set. We therefore turned to mixed logistic effects models to assess whether naming accuracy differs systematically for primary and secondary colour words.

Models were fitted using the lme4 package in *R*. We first fitted a model predicting colour naming accuracy on each trial for every patient as a function of the overall degree of anomia (as assessed by a standard picture-naming task) and the log word frequency as estimated from the BNC corpus. Both factors were treated as fixed effects; separate intercepts and slopes were fitted for each patient, with patient treated as a random effect. Model statistics are shown in Table 3: both the severity of the anomia and the log word frequency contributed significantly to the prediction of colour-naming accuracy.

We then fitted a second model adding colour type (primary/secondary) as an additional fixed effect, and compared the model fits. Including colour type significantly improved fit relative to the base model (*X*^2^=16.1, *p*<0.001). Significance tests on parameters of the elaborated model show that the effect of word frequency is no longer statistically reliable when colour type is included. The analysis thus appears to show a categorical difference in naming of primary versus secondary colours, over and above any effects of word frequency. Panel D in Fig. 3 shows the expected accuracy as a function of overall severity according to this model, plotted separately for primary and secondary colours. The lines show the expected performance for words with a mean log frequency, while the shading shows the range expected from the lowest to the highest frequency word.

Errors consisted mainly of semantic errors in which patients produced the wrong colour name, including “red,” “pink,” or “yellow” for orange; “blue” or “brown” for grey; “light red” for pink; and “yellow” for brown. Thus the naming data are consistent with, and also extend, the match-to-sample data. In both cases performance in SD was normal near red, green, and blue centers, and abnormal for colours between these regions (e.g. orange). Colours tested in the naming task but not in the match to sample task also showed that primary colours (including yellow, white, and black) were preserved whereas secondary colours (brown, gray, orange and pink) were seriously degraded. Together the results suggest that colour cognition in SD is largely preserved for the poles of the opponent-process colour space, but is disturbed for regions between the poles.

## 6 General discussion

In three tests, patients with SD exhibited a disorder of colour cognition in which (i) the ability to discriminate similar colours is preserved but the ability to group discriminably different colours into categories is disrupted, (ii) perception and short-term memory are preserved for spectra with peaks around 650 nm (red), 550 nm (green) and 450 nm (blue), but are degraded for spectral midpoints between these centers, and (iii) basic colour naming is preserved for primary colours—that is, the chromatic unique hues red, green, blue, yellow, and the achromatic polar colours black and white—but is impaired for secondary colours.

To our knowledge, this pattern of disruption has not previously been observed in any neuropsychological disorder. Cortical achromatopsia—the loss of colour vision attributable to lesions in regions of visual cortex that support processing of colour information—is a well-known phenomenon but does not produce the pattern of sparing and impairment seen in the current work and does not typically involve damage in anterior temporal regions (Bouvier and Engle, 2006). Case studies of colour perception in patients with aphasic syndromes have been previously reported, but paint a somewhat mixed picture. Some studies have described patients who appear to lose the ability to name colours, but with the ability to categorize and otherwise accurately discern similarities amongst colours relatively intact (e.g. Haslam et al., 2007). Others have described patients for whom an inability to name colours or understand colour words is observed coincident with abnormal colour sorting, but preserved colour discrimination (Roberson et al., 1999). Still others have suggested that colour names can be selectively preserved in the context of the general anomia observed in SD (Robinson and Cipolotti, 2001). A central challenge for disentangling these findings is that prior studies have tended to use quite heterogeneous methods across a variety of patient aetiologies, making it difficult to identify systematic trends across tasks and disorders. To our knowledge, the current work is unique in studying colour cognition across multiple tasks in a case series of patients with the same underlying cognitive disorder from the same aetiology.

### 6.1 Implications for theories of colour cognition

Perhaps most striking are the observed differences between patients and healthy controls in the match-to-sample task. The control behavior was largely consistent with classic studies of the human ability to discriminate different wavelengths of monochromatic light across the visible spectrum (Wright and Pitt, 1934). Such studies routinely find that the just-noticeable-difference (i.e., the difference needed to distinguish two stimuli with 75% accuracy) between two wavelengths of equal intensity is comparatively large (about 4 nm) for wavelengths near 460 nm (blue/indigo), 530 nm (yellowish green), and at either end of the visible spectrum (violet/red), and is comparatively small (less than 2 nm) in regions with local minima between these peaks at ~440 (purple), ~480 (blue/green), and ~575 (yellow/orange) nm (Long et al., 2006; Wright and Pitt, 1934). Accordingly, we found that controls were least accurate at finding the target among neighboring distractors for nonspectral colour samples with peak wavelengths near red (~650 nm), green (~550 nm), and blue (~450 nm) colour centers, and were most accurate at sample points between these regions, both in simultaneous and delayed paradigms. In contrast, patients with SD performed well in the red, green, and blue regions where controls were poorest, and showed worst performance in between these regions where controls were best.

This finding is of interest partly because it suggests that perception of colour similarity does not depend solely on early visual processing. Explanations of the hue discriminability curve in normal human vision are typically based on assumptions about neuronal interactions very early in the visual processing stream, before visual input even reaches cortex (Pokorny and Smith, 2004). The pathology in SD certainly does not influence very early visual processing (Nestor et al., 2006; Guo et al., 2013), nor is the documented pattern observed in other varieties of colour agnosias or cortical achromatopsias (Davidoff, 1991), The implications seem to be that healthy colour perception profiles reflect contributions of cortical processing in relatively anterior parts of the temporal lobes. What then accounts for these patterns? We here consider three different possible explanations.

1. *The effects arise from colour anomia*. One interpretation of the current findings is that they reflect knock-on effects of a general anomia. Recent research from a variety of labs has suggested that colour percepts are partly shaped by knowledge of colour names, either via learning or through cross-modal feedback from word representations to percepts (the “label feedback hypothesis”; see Lupyan, 2012). It is possible to view results from all three studies as arising from such factors. As previously noted, patients with SD may fail the Categorization oddball task because they are unable to retrieve the colour name associated with the target. Though the match to sample task does not demand overt naming, nevertheless colour perception and memory may be systematically altered when colour names are lost, by virtue of the loss of re-entrant feedback from labels that are automatically activated in healthy cognition. The colour naming data, of course, directly indicate anomia for colours (coincident with the general anomia that characterizes the disorder) in which lower-frequency colour names are more vulnerable than higher-frequency names. The greater disruption of perception and short-term memory for colours near spectral midpoints might thus arise because these are precisely the areas where the more vulnerable lower-frequency colour words have been lost.

Under this interpretation the results are of interest because they lend support to the still-controversial position that knowledge of colour names influences colour cognition. There are, however, some problems with this interpretation of our results. Most obviously, the account does not explain why naming of primary colours is so much more robust than naming of secondary colours, over and above effects of word frequency. Additionally, the impairments in colour-matching documented in Experiment 2 are not observed in patients with colour anomia from other aetiologies (e.g. Roberson et al., 1999; van Zandvoort et al., 2007). If the current phenomena arise solely because knowledge of colour words is lost, then it is unclear why different patterns of impairment are observed in other patients with colour anomia. Third, it is not clear why the loss of colour names at spectral midpoints would lead to a pattern in which perception and memory are systematically better at spectral colour centers. To see this, consider the following explanation of the control data. Samples and distractors near the red, green, and blue centers are perceptually very similar and all receive the same label. Samples near the midpoints are perceptually distinct and receive different labels (for instance, orange, yellow, green). When the sample and distractors all generate the same label, they are even more difficult to discriminate and it is hard to remember how they differ. When they all generate different labels, they are easier to discriminate and it is easier to remember how they differ. Thus performance excels near the midpoints and is worse near the centers. Patients who have lost the labels no longer get the benefits of improved discriminability and memory. Such an account explains why patients are impaired at midpoints, but not why they are better than controls at colour centers. Since the samples and distractors at the colour centers all receive the same label, retention of the label should continue to make these items more difficult to discriminate and remember.

2. *The impairments arise from damage to a ventral temporal region specialized for representing colour concepts*. This proposal accords with a series of brain imaging studies which together suggest that ventral temporal regions considerably anterior to the classical colour-processing occipital regions are engaged whenever participants must make difficult judgments on the basis of colour perception (Beauchamp, 1999; Simmons et al., 2007) or are required to retrieve the colours of familiar items (Chao et al., 1999; Chao and Martin, 1999; Kellenbach et al., 2001). To explain such findings, Simmons et al. (2007) proposed a region dedicated to the representation of “meaningful, high-level colour perceptual representations” (p. 2809) located approximately midway down the length of the fusiform gyrus (*y*=–33 to –38 in Talairach coordinates). If such a center contributes to perception, memory, and naming of colours, the SD deficits observed here might be caused by damage to this region.

A full assessment of this hypothesis would require a detailed study of the relationship between the distribution of atrophy in individual patients and the extent of their disordered colour cognition. In the current study, although basic structural MRI scans were of course conducted for diagnostic purposes, more extensive neuroradiology for some of the cases was either not carried out or was obtained at time-points well before or after the patients’ participation in these experiments. We thus do not have available, for a number of the cases, the kind of detailed assessment of the anterior-to-posterior extent of temporal-lobe atrophy at the time of the study that would be required to judge whether this hypothesis could account for our results.

It is also worth noting that the hypothesized region for colour concepts lies on the edge of the ventral temporal region susceptible to signal dropout by virtue of magnetic field inhomogeneities that arise from proximity to the air-filled sinuses. Without correction for these inhomogeneities, functional activations that extend anteriorly down the length of the temporal lobes may appear to be localized to a more posterior location (Devlin et al., 2000; Embleton et al., 2010; Lambon et al., 2010). Thus a further possibility is that this region is the posterior tail of a more anterior activation peak.

3. *Colour cognition is influenced by interactions within a grounded conceptual knowledge system*. The third hypothesis, and the one that we endorse, is that the observed effects arise because general knowledge about the world is “embodied,” that is, grounded in the same sensory and motor systems that govern our direct interaction with the environment (Barsalou et al., 2003; Martin, 2007; Patterson et al., 2007; Pulvermueller, 1999). On such theories, conceptual knowledge arises from the learned associations among different kinds of sensory, motor, and linguistic representations. The concept “stop-sign,” for instance, may include representations of octagonal shape in ventral temporal cortex; the colour “red” in area V4; phonological and orthographic codes for the words “stop-sign” in perisylvian regions; motor plans associated with stop-signs in parietal and premotor cortex; and learned associations among these. Many such theories propose that the network is interactive, so that activation of any of these sensory, motor, or linguistic representations promotes activation of associated representations in other modalities (Rogers et al., 2004). Thus the perceptual, motor, and linguistic representations—including perceptual representations of colour—can be influenced both by bottom-up input from sensory systems and by recurrent feedback from the rest of the semantic network. On this view, the degree to which different wavelengths of light appear similar depends, in the healthy system, both upon the structure present in the bottom-up signal and on recurrent interactions with other representations in the system via learned associations. This idea accords well with the many examples of knowledge-perception interactions recently reviewed by Collins and Olson (2014).

In SD, we propose that the influence of recurrent feedback on colour percepts is weakened or distorted, causing changes to the representation of colour in posterior cortex, especially for spectral regions where structure in the bottom-up signal is least robust (Lambon Ralph et al., 2007; Patterson et al., 2007; Rogers et al., 2004). It may be that, in regions of the spectrum corresponding to primary colours, the cortical states produced by bottom-up input alone are quite similar to those that arise from recurrent interactions within the healthy system, so that when these interactions degrade with disease, colour perception and naming is not compromised. In contrast, the cortical states that correspond to perception of secondary colours in the healthy system may depend more upon interactions within the cortical semantic network for their stability, so that when such interactions degrade, these states are more prone to drift—thus disrupting naming and altering perception of the corresponding colours (Goldstone, 2006).

Our reasons for preferring this hypothesis stem from prior studies of SD showing that, across a variety of disparate cognitive subdomains, the patients exhibit remarkably similar patterns of preservation and impairment (Lambon Ralph & Patterson, 2008; Patterson et al., 2006). Generally speaking, knowledge about statistical regularities exhibited within a particular modality of reception or expression tend to be spared in the disorder, while knowledge about properties of familiar items that violate such regularities tend to be seriously degraded. We have already seen how, in delayed-copy drawing, patients with SD omit properties that individuate related items (such as the hump of a camel, which distinguishes it from other animals) but retain properties shared by related items (such as the eye of a camel, which is common to animals). Similar patterns are observed in many other aspects of behavior. Patients with SD successfully retrieve the colours of everyday items when these are typical of the domain (e.g. the fact that bears, like many animals, are brown while cucumbers, like many plants, are green), but not when they are atypical (e.g. the fact that flamingos are pink or that carrots are orange; Rogers et al., 2007). They can accurately recognize words composed of orthographically well-formed letter strings (e.g. ROT), but fail to recognize words with unusual spellings (e.g. YACHT) and indeed may choose YOT in preference to YACHT as the real word (Rogers et al., 2004). They correctly read aloud words with typical spelling-sound correspondences (e,g., FEW), but very often regularize the pronunciations of those with atypical correspondences, thus pronouncing SEW as “sue” (Graham et al., 1994; Patterson and Hodges, 1992). They can generate the appropriate past-tense forms of regular verbs (such as blink → “blinked”), but are impaired with irregular forms (producing drink → “drinked”) (Patterson et al., 2001). In object-directed action, patients show disproportionate loss of unaffordanced action elements (Bozeat et al., 2002).

In many of these subdomains others have argued that the affected behavior—delayed-copy drawing, object recognition, object colour knowledge, word recognition, reading, spelling, verb inflection, and object-directed action—is supported by its own task-specific subsystem. Such a position requires, however, that many otherwise unrelated abilities are all simultaneously affected in similar ways by pathology in SD. This seems unlikely given the relatively circumscribed atrophy in the disorder and the additional observation that other forms of dementia, such as Alzheimer’s disease, that produce much more widespread brain abnormalities do not produce the same pattern of dysfunction (Nestor et al., 2006).

In prior work we have argued an alternative position, that the ability to retrieve irregular or atypical properties−properties of familiar items that violate the surface regularities of a given modality of representation−depends upon interactions between the modality in question and a more amodal representation of the item (Patterson et al., 2006). For instance, in the well-known “triangle” model of word reading, pronunciation of words with atypical spelling-sound correspondences depends upon input from the semantic representation of the word, because the direct pathway leading from orthography to phonology strongly encodes the systematic correspondences between spelling and sound that characterize regular words (Plaut et al., 1996). Spelling-sound pairings that violate this structure are difficult to process within this pathway and so come to depend more on additional input from representation of the word’s meaning. Similar models have been advanced to explain the effects of degraded semantic representation on memory for exceptional properties or items in the domains of delayed-copy drawing (Rogers et al., 2004) and verb inflection (Joanisse and Seidenberg, 1999). The models suggest that, when semantic representations degrade, their inputs to modality-specific representations throughout cortex are diminished or degraded. Since it is the irregular or atypical properties of items that depend most on this input for their activation, this loss mainly affects knowledge of such properties. This view thus explains impairments across many different tasks with reference to the same underlying cause, and further explains the particular *pattern* of deficit observed in each case. Our account of the current data accords well with this general view.

This view is also consistent with influential work in the development of colour knowledge conducted by Petzold and Sharpe (1998), who assessed short-term memory for colour samples using a delayed match-to-sample procedure in young children (3–6 years), older children (9–11), and young adults (22–30). In this case the stimuli were coloured circles against a white background, with colours expressed via non-spectral light with a carefully controlled dominant wavelength. From adult judgments, the authors measured the location of focal examples of blue, green, and yellow, as well as boundaries between these, and chose samples spanning the range from the blue/green to the green/yellow boundary that varied in dominant wavelength while controlling luminance. The task required participants to choose the sample match from an array of 16 different colours after a 5 s delay, allowing the authors to measure, not just accuracy, but the mean distance of the participant’s choice from the correct option.

**Fig. 4:**
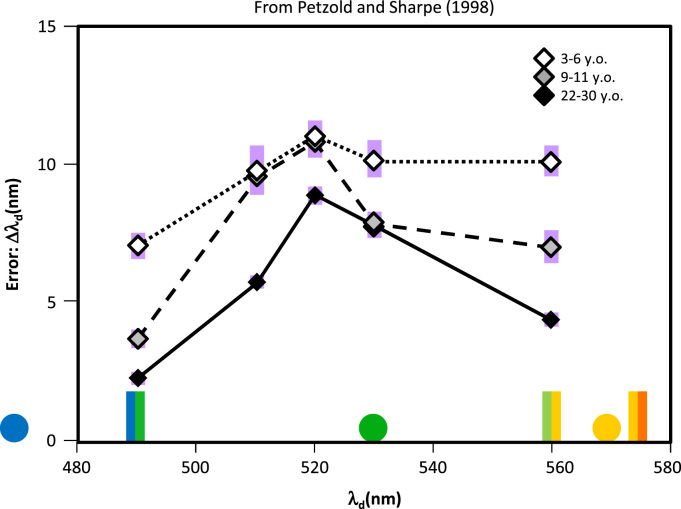
Development of short-term memory for colours. Data reported by Petzold and Sharpe (1998) in their study of the development of colour representations. The curves show mean spectral distance from the correct choice (ie error) in a 5 s delayed match-to sample task conducted with young children (3-6 years old), older children (9-11) and young adults (22-30). Circles show the location of adult focal colour judgments for blue, green, and yellow, while bi-coloured lines show the location of boundaries between these categories. Violet bars indicate standard error of the mean. As in the current study, performance is worst near the category center and best near the boundaries. Performance overall improves with age, but the extent of the improvement is larger near the boundaries than near the center, consistent with the view that learning and experience more strongly impact representations of colour near hue category boundaries. Redrawn with alterations from Petzold and Sharpe (1998) (For interpretation of the references to color in this figure legend, the reader is referred to the web version of this article.).

With these finely controlled stimuli, the authors observed essentially the same pattern noted for control participants in the current work: across age groups, sample memory was best at the hue category boundaries and worst near the category centers (Fig. 4). They also observed, however, improvement with increasing age that was most pronounced near the colour boundaries and least pronounced near the colour centers. Moreover, the age groups did not differ in their ability to detect small differences in dominant wavelength across this range of the spectrum in an oddball task similar that employed here. The data thus suggest that learning and experience between young childhood and adulthood, while not affecting the ability to detect subtle hue differences, nevertheless shape representations of colour, especially near hue category boundaries. These developmental patterns mirror the current data for SD patients, in which dissolution of knowledge about the world does not affect colour discrimination but erodes colour perception and memory for regions of the spectrum near hue category boundaries—that is, for secondary colours.

The idea that secondary colour categories are more strongly shaped by interactions with the knowledge system is also consistent both with classic evidence about the constancy of colour cognition across cultures and languages (Berlin and Kay, 1969; Heider, 1972; Kay, 2005) and with more recent evidence about the malleability of colour cognition through language and experience (Goldstone, 2006; Roberson et al., 2004; Roberson et al., 2000). The six colour categories that appear most robust to semantic impairment in SD—black, white, red, green, blue, and yellow—are precisely those that Berlin and Kay (1969) showed are most likely to be adopted consistently across different languages and cultures. They also correspond roughly to the poles of the three-dimensional opponent-process colour space, which are thought to be carried by separate subcortical and cortical channels in the human visual system (Solomon and Lennie, 2007). Thus the current evidence supports the long-standing view that these colour categories are inherent in the bottom-up structure provided by our perceptual systems, and do not depend strongly on learning and experience. Instead, such effects may have their strongest impact on colour concepts existing between the poles of the opponent-process colour space.

In this, the data are consistent with the variety of cross-linguistic and developmental studies showing that linguistic experience can alter perceptual boundaries between colour categories (Roberson et al., 2004; Roberson et al., 2000). Indeed, as Kay and Regier (2003) have noted, such studies appear to show their principal effects at the boundaries between colour categories. In contrast, colour foci (i.e., the regions of colour space judged as “best examples” of a given colour) do not appear to vary with linguistic or other experience. Both observations are consistent with the view that the primary colour centers are strongly shaped by bottom-up input, but that the perceptual regions between these centers, and the mappings from these regions to explicit labels, can be influenced by learning and experience—that is, by recurrent interactions within the conceptual knowledge system. The conclusion most consistent with all of these findings is that knowledge does shape perception, but only within limits.

## 7 Author contributions

Rogers designed and programmed the computer-based tasks and conducted all data analyses. Graham and Patterson designed the colour-naming task, recruited the patients and conducted the patient testing with the help of research assistants. All authors contributed to design of the experiments and the writing of the manuscript.

## Figures and Tables

**Table A1:** Parameters for the red, green, and blue colour filters for target and distractor items in the oddball task. *Closest item* indicates the Euclidean distance between the target and the closest distractor in each array. Items in the Categorization and Discrimination conditions are paired such that the minimal parameter distance in the Categorization condition is always larger than the minimal distance in the paired Discrimination condition. Cat=Categorization item, Disc=Discrimination item.

Item no.	Condition	*Target*		*Distractor* 1			*Distractor* 2			*Distractor* 3			*Closest*	
		**r**	**g**	**b**	**r**	**g**	**b**	**r**	**g**	**b**	**r**	**g**	**b**	*item*
1	Cat	0	128	106	0	101	138	0	59	91	0	101	138	42
2	Cat	137	106	0	89	112	0	0	115	0	58	141	0	48
3	Cat	170	74	93	182	38	125	108	0	119	134	23	96	50
4	Cat	0	104	135	22	101	85	18	101	17	0	132	54	55
5	Cat	20	204	204	27	188	19	22	219	150	17	174	64	56
6	Cat	20	204	204	78	160	18	24	145	16	22	219	150	56
7	Cat	169	225	86	178	162	79	255	216	114	255	119	93	64
8	Cat	153	46	105	181	132	54	139	45	42	196	64	59	65
9	Cat	171	148	27	102	143	35	23	191	134	19	215	44	70
10	Cat	129	169	11	222	203	15	215	67	14	162	104	10	73
11	Cat	33	144	164	83	193	39	45	225	102	28	138	87	77
12	Cat	234	70	174	188	74	56	250	75	90	204	156	61	86
13	Cat	80	150	0	206	20	0	211	194	0	145	94	0	86
14	Cat	46	230	230	68	230	60	25	173	120	118	236	153	106
15	Cat	0	130	190	0	0	190	91	0	228	55	60	255	110
16	Cat	242	202	57	74	182	106	144	238	113	132	185	32	114
17	Cat	132	230	34	230	139	22	230	82	82	230	220	94	115
18	Cat	230	46	230	230	117	91	230	19	79	230	213	64	153
1	Disc	46	99	128	47	119	128	47	119	128	47	119	128	20
2	Disc	56	128	39	31	128	46	31	128	46	31	128	46	26
3	Disc	11	56	110	12	91	115	12	91	115	12	91	115	35
4	Disc	0	24	101	0	59	91	0	59	91	0	59	91	36
5	Disc	128	47	48	128	51	88	128	51	88	128	51	88	40
6	Disc	106	128	21	88	128	60	88	128	60	88	128	60	43
7	Disc	180	161	255	226	168	255	226	168	255	226	168	255	47
8	Disc	27	188	19	17	174	64	17	174	64	17	174	64	48
9	Disc	230	152	129	230	202	132	230	202	132	230	202	132	50
10	Disc	106	230	170	125	230	121	125	230	121	125	230	121	53
11	Disc	181	107	54	196	59	81	196	59	81	196	59	81	57
12	Disc	37	149	0	0	122	35	0	122	35	0	122	35	58
13	Disc	68	230	60	73	230	120	73	230	120	73	230	120	60
14	Disc	33	122	230	40	182	230	40	182	230	40	182	230	60
15	Disc	125	159	16	66	144	14	66	144	14	66	144	14	61
16	Disc	230	213	64	230	117	91	230	117	91	230	117	91	100
17	Disc	225	110	68	233	219	70	233	219	70	233	219	70	109
18	Disc	230	82	82	230	220	94	230	220	94	230	220	94	139

**Table 1:** 

	*Control*Mean (SD)	*Patients with semantic dementia*									
		Ath	NS	WM	AN	Ate	JM	IB	RS	FB	JR
*Demographic*											
Age		64	71	57	66	69	66	58	57	72	58
Sex		M	F	F	M	M	M	M	M	F	M
Education		10	9	16	9	20	15	11	16	16	20

*General neuropsychology*											
MMSE	28.8 (0.9)	**5**	**8**	**19**	26	**1**	24	**23**	**18**	**23**	**20**
ACE	93.8 (3.5)	**9**	**12**	**30**	**56**	**5**	**48**	**40**	–	**63**	**29**

*Visual perception*											
Rey-Copy /36	34.2 (1.6)	**21**	34	36	35	36	36	32	29	36	36
VOSP Cubes /10	9.3 (1.5)	**4**	10	9	10	10	9	9	10	9	–
VOSP Dot count /10	9.9 (0.3)	10	9	9	10	10	10	10	10	10	9
*Memory*											
Forward digit span	7.1 (0.9)	5	4	5	6	7	7	8	7	7	8
Backward digit span	5.4 (1.4)	**2**	3	4	4	5	7	5	6	5	6
Rey-Delayed copy /36	18.3 (5.2)	0	0	12	9	8	17	4	11.5	10.5	–

*Semantic*											
WPM	0.99 (0.01)	**0.19**	**0.02**	**0.28**	**0.63**	**0.45**	**0.95**	**0.30**	**0.34**	**0.84**	**0.33**
Naming	0.97 (0.02)	**0.05**	**0.02**	**0.28**	**0.38**	**0.08**	**0.47**	**0.08**	**0.03**	**0.16**	**0.09**
PPT-Pic	0.98 (0.03)	**0.06**	**0.75**	**0.85**	**0.77**	**0.46**	**0.88**	**0.63**	**0.73**	**0.90**	–

*Note*: Bold font indicates impaired performance according to published cutoffs. Dashes indicate that no data were available for the corresponding test within a year of completing the colour battery. MMSE = Minnesota Mental-State Exam; ACE = Addenbrooke's Cognitive Examination; VOSP = Visual Object and Space Perception; PPT = Pyramids and Palm Trees Test.

**Table 2:** Demographic and neuropsychological data for 12 patients in Experiment 3.

		ControlsMean (SD)	AN	WM	ATe	KH	MA	JCh	JCh	SL	MS	JL	DG	JH
*Demographic*														
Sex			M	F	M	M	M	M	F	F	F	M	F	F
Age			63	52	65	57	63	58	57	52	68	62	62	58
Education			9	19	19	14	15	10	10	10	17	9	10	9

*General neuropsychology*														
MMSE		28.8 (0.9)	30	24	25	**23**	29	**19**	**20**	24	—	24	**20**	24
Visual perception														
Rey copy		34.2 (1.6)	36	36	36	33	36	31	32	28	—	34	25.5	31
VOSP cubes (/10)		9.3 (1.5)	10	10	10	10	10	10	—	10	**4**	7	**5**	10
VOSP dot count (/10)		9.9 (0.3)	10	—	10	10	10	9	—	10	10	10	10	—
													
*Memory*														
Digit span forward		7.1 (0.9)	7	8	8	6	6	7	6	5	—	7	6	7
Digit span backward		5.4 (1.4)	7	5	4	4	3	4	4	4	—	4	3	4
Rey delayed copy		18.3 (5.2)	26	25	24	13.5	6.5	12.5	12.5	14	—	**3**	**3.5**	12
												
*Semantic battery (proportion correct)*														
Word-picture matching	0.99 (0.01)		1.00	0.98	**0.91**	**0.91**	**0.89**	**0.88**	**0.85**	**0.75**	**0.54**	**0.52**	**0.52**	**0.50**
Naming	0.97 (0.02)		1.00	**0.41**	**0.16**	**0.77**	**0.20**	**0.64**	**0.06**	**0.28**	**0.02**	**0.17**	**0.19**	**0.16**
PPT-words	0.98 (0.02)		**0.92**	0.96	**0.85**	0.96	**0.77**	—	**0.85**	**0.73**	**0.52**	**0.52**	**0.56**	**0.60**
PPT-pics	0.98 (0.03)		0.98	**0.85**	**0.90**	**0.87**	**0.83**	—	**0.81**	**0.87**	**0.60**	**0.50**	**0.40**	**0.63**

*Note*: Bold indicates impaired performance according to published cutoffs. Figures in shaded cells were taken from an older version of the corresponding test. Dashes indicate that no data were available for the corresponding test within a year of completing the colour battery. MMSE=Minnesota Mental-State Exam; VOSP=Visual Object and Space Perception; PPT=Pyramids and Palm Trees Test.

**Table 3:** Logistic mixed effects model fits and comparison.

*Model* 1 *fixed effects*	*B*	*Serr*	*Z*	*p<*	*Sig*
Intercept	−8.8	2.4	−3.7	0.001	⁎⁎⁎
Object naming accuracy	3.7	1.6	2.4	0.02	⁎
Log word frequency (BNC)	2.3	0.6	3.9	0.001	⁎⁎⁎
					
*Model* 2 *fixed effects*					
Intercept (Colour type: Primary)	−0.3	3.6	-0.1	0.930	
Object naming accuracy	4.1	1.6	2.5	0.020	⁎
Log word frequency (BNC)	0.8	0.8	1.0	0.300	
Colour type: Secondary	−3.9	1.3	-3.0	0.003	⁎⁎
Fit comparisons	*AIC*	*BIC*	log* lik*	*deviance*	*Sig*
Model 1	92	108	-40	80	
Model 2	78	97	−32	64	
Comparison	*X*^2^	=16	*p*<0.0001		⁎⁎⁎

*NOTE*: Separate intercepts and word-frequency slopes were fit for each patient, with these treated as random effects. AIC=Akaike information criterion; BIC=Bayesian information criterion; log lik=model log likelihood.

## Appendix

*Hardware details and stimulus parameters for* Experiments 1 and 2.

The LCD monitors employed cold-cathode fluorescent backlighting. This technology produces white light with multiple spectral peaks, bandpass-filtered at each pixel through three immediately adjacent twisted nematic devices (“liquid crystals”). Each such device serves as a filter controlling the passage of light in a given frequency band, and the spectral structure of the backlight together with the three filters produce light with separately-controllable peaks around 650 nm (red), 550 nm (green) and 450 nm (blue). When fully open, each filter permits passage of light within its bandwidth at approximately equal peak intensities, and when fully closed each filter fully blocks passage of light in its bandwidth. Each filter is voltage controlled with an 8 bit gate, allowing a total of 255 different states corresponding to 255 monotonically increasing intensities of light passing through each filter. The computer software thus specifies colours by directly setting the state of each of the three filters at every voxel to one of 255 possible values. For instance, the values 255, 0, 0 would fully open the 650 nm filter, allowing passage of light with a single spectral peak near 650 nm, typically perceived as red. The values 255, 255, 0, would fully open both the 650 nm and 550 nm filters, producing light with spectral peaks at both 650 nm and 550 nm, typically perceived as yellow.

The colour parameter settings used for the target and distractor items for Experiment 1 are listed in Table A1. For Experiment 2, the stimuli were generated as follows. The spectrum began with the 650 nm filter at each pixel fully open and the other two filters fully closed (255,0,0 in the filter parameter space), a point henceforth referred to as the “red center.” Colours shifted more toward the blue end of the spectrum were generated by keeping the red filter open and opening the green filter (~550 nm) in increasing increments until both it and the red filter were fully open, a point henceforth referred to as the red–green midpoint. Shifts further toward the blue end of the spectrum were created by diminishing the red filter until it was fully closed, leaving only the green filter fully open (“green center”); then gradually opening the blue filter (~450 nm) until both green and blue were fully open (green–blue midpoint). Moving still further toward the blue end, the green filter was gradually diminished until it was fully off, leaving just the blue filter fully open (“blue center”); and finally to generate colours in the violet part of the spectrum, the red filter was incrementally opened up until both red and blue were maximally open (blue–red midpoint), and then the blue filter was incrementally reduced until only the red filter was fully open, returning to the origin. In this way we created the fully saturated and luminant spectrum from which samples and distractors for Experiment 2 were selected as described in the text.
